# Food and the brain: Neural and endocrine control of feeding, metabolism, and reproduction

**DOI:** 10.1111/jne.70104

**Published:** 2025-10-28

**Authors:** Naira da Silva Mansano, Calvin Vinh Lieu, Alfonso Abizaid

**Affiliations:** ^1^ National Institute of Neurological Disorders and Stroke Bethesda Maryland USA; ^2^ Department of Physiology University of Toronto Toronto Ontario Canada; ^3^ Neuroscience Department Carleton University Ottawa Ontario Canada

**Keywords:** energy balance, ghrelin, GnRH, leptin, reproduction

## Abstract

Feeding and reproductive function are regulated by intricate systems that monitor food availability and energy stores, and on the basis of energy status, promote or put a brake on reproduction. This is particularly evident in the systems that regulate feeding and reproductive state in female mammals. Here we describe some of the systems that regulate feeding and reproductive state focusing on how metabolic hormones impact the onset of puberty as discussed in the panel session presented at the recent Panamerican Neuroendocrine Society meeting in Santos, Brazil. Indeed, hormones like leptin and insulin, which are released when levels of energy resources are increasing, may be critical signals that activate hypothalamic pathways related to ovulation in females to cause the onset of puberty. In adults, increasing levels of these hormones signal to the hypothalamus to reduce food intake and increase energy expenditure. In contrast, hormones like ghrelin impact hypothalamic and extrahypothalamic brain regions to drive hunger and the motivation to eat ultimately increasing feeding behavior and decreasing energy expenditure. Based on these actions, we describe some potential targets for the treatment of obesity and the mechanisms by which these targets work to improve human health.

## 1 INTRODUCTION

The regulation of feeding and energy balance is orchestrated by the integration of internal and external signals that allow organisms to eat not only when they are low in circulating nutrients but also when environmental cues predict the presence of foods, or the future scarcity of food resources as it occurs in seasonal species. As part of this regulation, organisms respond to these signals in ways that maximize survival at the expense of reproductive efforts.^1^ This is particularly important in female mammals where the investment in caring for offspring when food sources are scarce can compromise not only the survival of their young but also their own survival.^1^ This exquisite orchestration of processes is attained through a complex network of brain regions that can modulate basic metabolic mechanisms such as energy expenditure, glucose utilization and feeding, and by connected networks important for more complex functions that include motivation, emotion and cognition, all required for successful procurement of food and potential mates, and avoidance of predators.^2^ Not surprisingly, reproduction in females from puberty to reproductive senescence, is highly sensitive to changes in the energy state, with reproductive function being inhibited in negative energy balance states.^1^ In this review, we review current topics discussed as part of a symposium presented at the Panamerican Neuroendocrine Society (PANS) 2024 meeting in Santos Brazil. The symposium included presentations related to current treatments for obesity and metabolic disorders, the hormonal regulation of hypothalamic and extrahypothalamic brain regions to increase food motivation, and the potential for metabolic signals to regulate the onset of puberty. Overall, these presentations reflect the complexity in the regulation of feeding and its impact on reproduction.

## 2 BRAIN CIRCUITS REGULATING ENERGY BALANCE AND REPRODUCTION

The hypothalamus and brain stem constitute two brain regions that serve to monitor internal levels of nutrients and energy stores.^3^ Cells within these regions lack a fully functional blood–brain barrier and are critical for conveying metabolic signals to the rest of the brain and regulating energy state.^4^ Amongst them are cells in the hypothalamic arcuate nucleus (ARC) that are part of what is known as the melanocortin system, and that include neurons that secrete anorectic peptides like α‐melanocyte stimulating hormone (α‐MSH), or orexigenic peptides like neuropeptide y (NPY) and the agouti‐related peptide (AgRP).^4^ These peptidergic cell groups target overlapping hypothalamic and extra‐hypothalamic brain regions to modulate appetite and energy expenditure, in some cases by acting on the same receptor. Indeed, α‐MSH and AgRP bind to melanocortin receptors 3 and 4 (MC3/4 receptors) with α‐MSH working as an agonist to decrease appetite and increase energy expenditure, while AgRP acts as an antagonist to increase appetite and reduce energy expenditure.^5^ Projections from NPY/AgRP and proopiomelanocortin (POMC) neurons are distributed throughout the hypothalamus where they can affect feeding, metabolism and reproductive function including hypothalamic regions like the paraventricular hypothalamus, the dorsomedial hypothalamus, and the lateral hypothalamic area (LH).^6^ In the LH, targets for NPY/AgRP and POMC neurons include cells that produce two peptides associated with homeostatic responses. The first set of cells includes neurons that produce the peptide orexin/hypocretin and the second set includes neurons that produce the melanocortin hormone (MCH) peptide. NPY/AgRP neurons stimulate the activity of orexin/hypocretin cells while POMC neurons inhibit these cells.^7^, ^8^ When activated, these neuronal cell groups produce hyperphagia, increase food motivation and regulate metabolic function but they do so through pathways that are separate from each other.^4^ Importantly, both orexin and MCH may integrate metabolic information to regulate reproduction.^9^, ^10^, ^11^

In the brain stem, sensory cells in the area postrema (AP) dynamically monitor changes in nutrients like glucose and fatty acids and rapidly respond to fluctuations in these nutrients.^12^, ^13^ These nutrient‐responsive cells project to the nucleus of the solitary tract (NTS), a brain stem region ventrally adjacent to the AP, and critical for receiving ascending information from the gut via vagus nerve afferent.^12^, ^13^ The AP and NTS also contain receptors for metabolic hormones like leptin, ghrelin, glucagon‐like peptide (GLP‐1) and multiple others, and project to forebrain regions that respond to rapid, acute changes in nutritional signals that include hyperglycemia, gut fullness, and increased nutrient absorption. In particular, GLP‐1, is a satiety signal released when nutrients are absorbed and that reduces appetite.^12^, ^13^, ^14^, ^15^ Indeed, the discovery of GLP‐1 and its receptors has been fundamental for the generation of the most successful drugs to curb obesity, type II diabetes and potentially other metabolic‐related disorders like kidney and cardiovascular disease.^16^, ^17^

## 3 METABOLIC FUELS FOR REPRODUCTION

There is substantial evidence that metabolic signals like glucose and fatty acids, as well as metabolic hormones, play an important role in the regulation of reproduction.^1^, ^18^ This is important especially in female mammals because they carry the greater metabolic investment in producing and caring for offspring during pregnancy and lactation.^1^, ^18^ Indeed, the reproductive system of female mammals is extremely sensitive to these signals and is inhibited during situations where nutrients are scarce.^1^, ^19^ This is particularly evident during milestone adult reproductive events like the onset of ovulation during adolescence, seasonal changes in food availability, the beginning of pregnancy, and lactation.^1^, ^19^ Not surprisingly, when nutrient availability is low, female mammals forgo reproductive efforts, to enhance survival.

Reproduction is regulated by the hypothalamic–pituitary–gonadal (HPG) axis, a system that includes sexually dimorphic neuroendocrine pathways.^20^, ^21^ In this system, gonadotropin‐releasing hormone (GnRH) is released from scattered neurons within the anterior preoptic area into the median eminence where the peptide is carried to the anterior pituitary to stimulate the release of luteinizing and follicular‐stimulating hormones (LH and FSH, respectively). These hormones stimulate ovarian cells to mature follicles and release steroid hormones like estrogen and progesterone. Among the critical actions of these steroid hormones is their ability to provide feedback signals to the hypothalamus to regulate GnRH release. In males, gonadal steroids produce a negative feedback signal that reduces GnRH release, but in females these hormones have both a negative and a positive feedback effect the latter required for ovulation to occur.^22^

**FIGURE 1:**
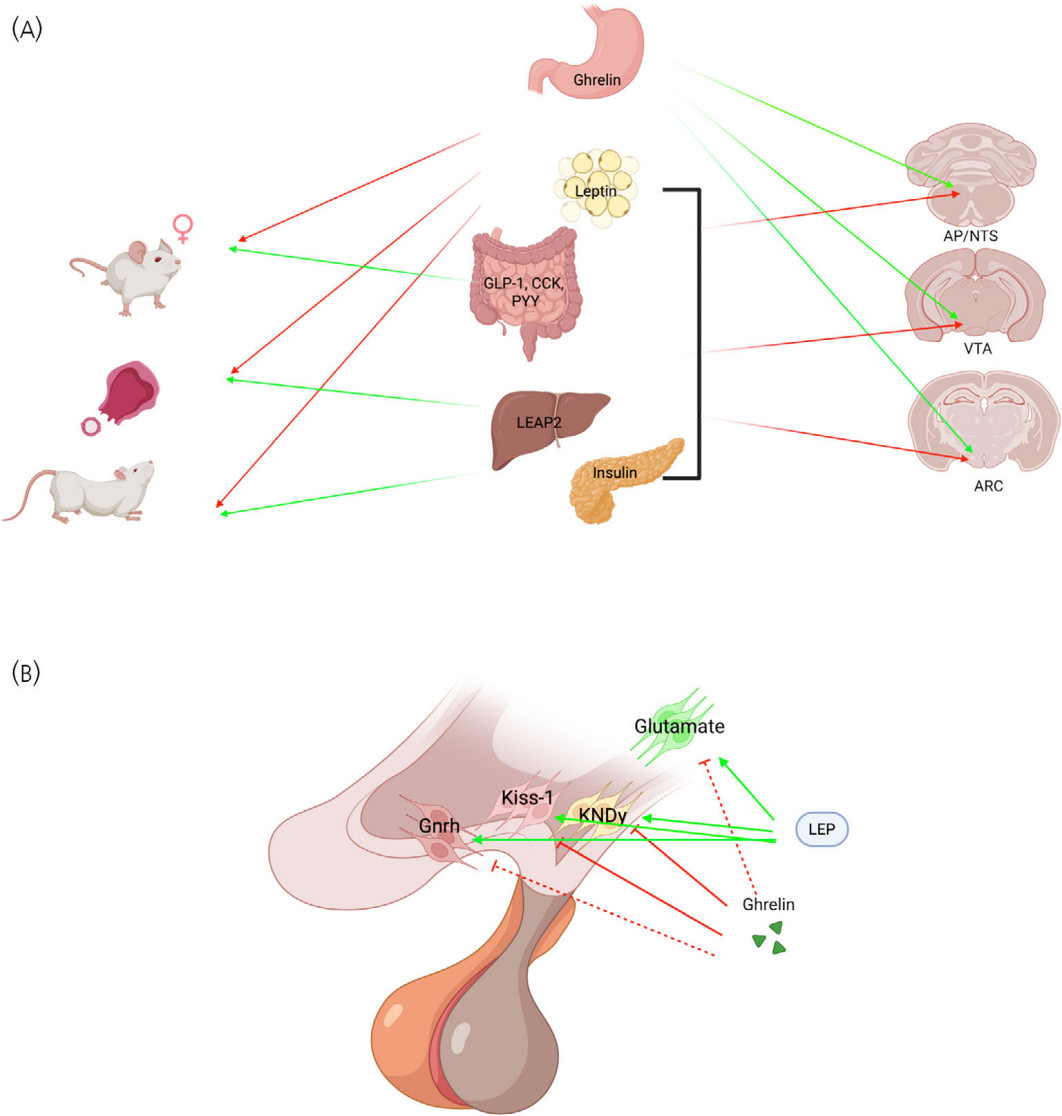
Metabolic hormone regulation of reproduction and energy balance. (A) How most satiety hormones including leptin, insulin and GLP‐1 act centrally to promote fertility and sex behavior, whereas ghrelin, a hormone that signals a negative energy state, reduces fertility and sex motivation. These effects may be mediated by the actions of these hormones on receptors located within the brain stem area postrema and nucleus of the solitary tract (AP/NTS), midbrain ventral tegmental area (VTA), and the preoptic and arcuate nucleus (ARC) of the hypothalamus. (B) How ghrelin and leptin can act across different regions of the hypothalamus to regulate the onset of puberty (glutamate cells in the ventral premammillary nucleus [PMV]), gonadotropin‐releasing hormone (GnRH) and luteinizing hormone (LH) pulsatility (neuropeptide y [NPY]/agouti‐related peptide [AgRP], proopiomelanocortin (POMC), and kispeptin 1/Neurokinin/dynorphin (KNDy) neurons in the ARC), or the LH surge (Kisspeptin 1neurons in the anteroventral preoptic area; anteroventral preoptic area [AVPV]). Dashed lines reflect putative effects.

The generation of the pro‐ovulatory GnRH and subsequent LH surges is mediated by the anteroventral preoptic area (AVPV), an anterior hypothalamic region that is larger in females than it is in males.^22^ A subset of neurons within the AVPV produces the peptide kisspeptin 1 (Kiss1), and these neurons regulate GnRH secretion.^22^ Importantly, in females, these neurons integrate information coming from ovarian hormones including estrogen and progesterone and release Kiss1 to stimulate the surge in GnRH and LH.^22^ A second group of Kiss1 neurons is found in the ARC. The ARC Kiss1 neurons also secrete neurokinin B and dynorphin and are often called KNDy neurons.^23^ These cells are not important for the GnRH/LH surges but play an important role in the control of pulsatile release of GnRH. Neurokinin D and dynorphin released from these neurons have an autocrine effect, with neurokinin D stimulating the release of Kiss1 while dynorphin reduces the release of Kiss 1 to produce pulses of GnRH secretion.^23^ Kiss1 neurons in the AVPV and KNDy neurons in the ARC respond to metabolic and endocrine signals^24^ and have been linked to the regulation of reproductive function by changes in energy balance at different periods of development (see Figure 1).^25^

Metabolic hormones and neuropeptides can influence circuits that regulate fertility so that reproduction only occurs when nutrient levels allow for a successful reproductive effort.^18^ Thus, signals like leptin, a key hormone that links nutritional status to puberty, are secreted in proportion to adiposity and signal the brain about the body's energy reserves.^26^, ^27^ Leptin levels are increased when animals have accumulated sufficient levels of fat stores, promoting the onset of ovulation in developing females, and gating ovulatory cycles in adult females.^18^ Moreover, sensitivity to leptin is decreased during pregnancy and lactation together with the inhibition of the reproductive axis during these states.^19^ Leptin deficiency, caused by either excessive leanness or genetic mutations, leads to delayed puberty in female mice^28^ and may result in hypogonadotropic hypogonadism, a condition that can be reversed with leptin treatment.^26^

The importance of leptin on female reproduction is highlighted by work showing leptin as one of the most important signals for the onset of puberty in mammals.^29^ The ability of leptin to advance puberty or prevent the impact of food scarcity on the delay of puberty, is mediated by several hypothalamic sites including the hypothalamic ventral premammillary nucleus (PMV), a nucleus found in the most‐posterior portion of the medial hypothalamus just caudal to the ARC, and one rich in leptin receptors.^29^, ^30^ Increasing leptin concentrations activate neurons in the PMV which activates AVPV Kiss1 neurons and GnRH neurons in the anterior preoptic area.^31^ With sufficient estradiol positive feedback these neurons activate GnRH cells to produce the LH surge required for the first ovulatory episode.

There is also evidence that feedback from melanocortin cells in the ARC can gate metabolic signals to directly influence GnRH electrical activity, secretion, and release.^32^ Food restriction or intracerebroventricular (ICV) infusions of NPY into the brain of prepubertal animals can delay the onset of puberty in rodents and primates, whereas treatment with α‐MSH can advance the onset of puberty.^33^, ^34^, ^35^ While most studies suggest that the effects of NPY and α‐MSH on the timing of puberty are mediated through KNDy neurons,^36^, ^37^ there is also evidence indicating that these effects occur independently of these neurons.^38^ Regardless of the involvement of KNDy neurons in the determination of the timing of puberty, it seems like the ARC integrates nutritional signals from a wide range of systems to maximize reproductive success when nutrients are available.^39^, ^40^, ^41^ Indeed, electrophysiological studies show that NPY inhibits the activity of KNDy neurons.^41^, ^42^ Additionally, other studies have demonstrated that NPY also directly inhibits GnRH neurons.^32^, ^43^ Furthermore, melanocortin receptor activation, specifically MC3 and MC4, along with α‐MSH, directly enhance the excitability of GnRH neurons in female mice.^32^ One emerging hypothesis is that KNDy neurons directly sense changes in energy homeostasis and regulate reproductive function.^44^, ^45^, ^46^ It remains unclear the degree to which specific ARC neurons are responsible for conveying nutritional status information to the reproductive axis and this search remains an exciting field to study with new genetic tool sets becoming available.

## 4 NEUROPEPTIDES AS TARGETS TO CURB OBESITY

Understanding the circuits governing the regulation of energy balance and reproduction is critical for the development of treatments that curb obesity and the consequences that this metabolic state can have on fertility. It is accepted that obesity is a complex condition that results from an interaction between biological, behavioral and environmental factors. Some of the primary causes of obesity include the excessive consumption of calorie‐dense diets and a sedentary lifestyle.^47^, ^48^ Thus, the first line of treatment for obesity constitutes lifestyle changes, that include alterations to diet and increased physical activity. Although simple, most patients placed on diets are only able to achieve modest reductions in weight and on average regain 79% of the weight lost within 5 years.^49^, ^50^ Surgical intervention in the form of gastric bypass is an effective means to lose weight but is limited to individuals with morbid obesity (Body mass index (BMI) > 40 or BMI > 35 with additional health conditions) and 73% of patients who undergo gastric bypass regain significant weight.^51^

For these reasons, the need for pharmaceutical interventions remains critical for the treatment of obesity and metabolic disorders. Given the role of NPY in increasing appetite, NPY was thought of as a potential target for obesity therapeutics. Hypothalamic NPY expression is increased following fasting and ICV injection of NPY into the third ventricle increases food intake dramatically.^52^, ^53^ Furthermore, NPY levels are elevated in the serum of obese women and both mRNA and protein are increased in the hypothalamus of obese rodents.^54^, ^55^, ^56^ Importantly, global knockout of NPY in mice is protective against high fat diet (HFD)‐induced obesity.^57^ Therefore, treatments that decrease NPY expression or block NPY receptor signaling may be effective in treating obesity. The orexigenic effects of NPY are driven by receptors NPY1R and NPY5R, both of which are G‐protein coupled receptors, in the hypothalamus. The first attempts to generate antagonists for NPY1R occurred in the early 1990s and although successful in targeting the receptor and suppressing food intake, their inability to penetrate the blood–brain barrier made their therapeutic potential limited.^58^, ^59^ In 2011, scientists at Pfizer and Neurogen demonstrated that their orally administered and blood–brain barrier permeable NPY1R antagonist was able to modestly decrease food intake in rats, but long‐term studies were not done to evaluate its weight loss potential.^60^ There has yet to be an NPY1R antagonist that has reached clinical trials. As for NPY5R, antagonism of the receptor was shown to significantly decrease food intake and body weight in rodent models, but this did not translate to clinically significant weight loss in humans.^61^, ^62^ Therefore, blocking an individual NPY receptor is unlikely to be sufficient to induce significant weight loss. Preclinical rodent studies show that combined NPY1R and NPYY5 receptor antagonism is effective in reducing food intake and weight gain,^63^ yet there have not been any studies that have evaluated the potential of simultaneously blocking these two receptors in human trials. Early studies into the modulation of NPY signaling as a means for obesity treatment have demonstrated some promise but there is still much to be done.

Despite advances in the use of compounds blocking NPY receptors, attention was diverted to the generation of drugs preventing the absorption of fatty acids, drugs that resulted in weight loss as a side effect, or drugs mimicking satiety signals from the gut. The current most common pharmaceutical interventions for obesity include orlistat, contrave, and GLP‐1 receptor (GLP‐1R) agonists like semaglutide. Orlistat is a lipase inhibitor that prevents the breakdown of triglycerides to free fatty acids and therefore impairs the absorption of lipids. Contrave is a combination drug made up of the norepinephrine/dopamine reuptake inhibitor bupropion, and the opioid receptor antagonist naltrexone, and a drug that increases the release of α‐MSH from POMC neurons.^64^ While both orlistat and Contrave can achieve modest weight loss (~6%) without lifestyle alterations, both have substantial side effects ranging from GI perturbations to increased risk for suicidal thoughts.^64^, ^65^ More recently, GLP‐1R agonists have emerged as the primary treatment for obesity and metabolic disorders. The first GLP‐1R agonist‐based drug approved by the FDA was exenatide in 2004 but recent advancements have brought about semaglutide and tirzepatide. These drugs improve upon exenatide with significantly longer half‐lives and prolonged presence in blood.^66^ In the case of tirzepatide, it also acts as a dual agonist for GLP‐1R and gastric inhibitory polypeptide receptor, which act synergistically to increase weight loss.^67^ GLP‐1R agonists act via a combination of mechanisms including the slowing of gastric emptying, suppressing appetite via actions in the brain, and increasing insulin secretion.^68^, ^69^, ^70^, ^71^ At their highest dosages semaglutide and tirzepatide induce 14.9% and 20.9% weight loss respectively, multiple times greater than what has been on the market.^72^, ^73^

Notably, obesity has been linked with alterations in reproduction. Generally, metabolic signals like leptin, insulin, GLP‐1 and other satiety hormones generally promote the activity of the reproductive axis. Nevertheless, sensitivity to some of these hormones (i.e., leptin, insulin) is reduced in obese individuals both peripherally and centrally, and that is associated with decreased fertility.^74^, ^75^ This has led to the hypothesis that treatments that improve metabolic function may also be helpful in improving fertility.^36^ For instance, GLP‐1 can directly activate the firing of GnRH neurons in mice,^37^ and direct infusions of GLP‐1 into the median eminence produce LH surges in sheep.^38^ Moreover, GLP‐1R are increased just prior to ovulation, and peripheral injections of GLP‐1 or its analog Exedin 4, augment the magnitude of the LH surge in female rats.^76^ Chronic central administration of GLP‐1 advanced puberty in female rats.^76^ Overall, these data support the notion that GLP‐1 or its agonists can improve fertility. While this approach has been used successfully to improve fertility in some forms of obesity (i.e., obesity associated with polycystic ovarian syndrome) it is less clear if these effects are due to the direct action of GLP‐1 agonists on the neuroendocrine systems that regulate energy balance and fertility or through actions on the periphery that, for instance, improve metabolic health via a reduction of inflammatory signals.^77^

Despite the effectiveness of GLP‐1R agonists on improving the metabolic (and potentially the reproductive) outcome of obese individuals, 82% of users report adverse gastrointestinal events and have a discontinuation rate as high as 25% with the strongest doses.^67^, ^78^ GLP‐1R agonist drugs face a similar hurdle as lifestyle alterations, as only 40% and 17% of patients remain on semaglutide and liraglutide respectively after 1 year and regain two‐thirds of their weight within a year of stopping the drug.^73^, ^79^ In addition, accumulating data suggest that chronic use of GLP‐1 agonists may have other serious side effects that include gastrointestinal malaise, nausea, vomiting, pancreatitis bone loss and abdominal pain.^80^ These negative side effects may be due to the central pathways that GLP‐1 agonists activate to decrease appetite, which include brain stem pathways associated with gastrointestinal malaise.^81^, ^82^ Although there have been many advancements in obesity pharmaceuticals, these still face difficult hurdles such as adverse side effects, persistence in their usage, and weight regain after withdrawal.

## 5 GHRELIN AND NON‐HOMEOSTATIC FEEDING

Treatments aimed at curbing obesity have focused on targeting homeostatic hormones given their actions on the hypothalamus and brain stem. One caveat to this is that many of the side effects produced by anti‐obesity drugs are side effects that include depression and anxiety, suggesting that metabolic signals and the drugs that mimic their metabolic effects also act on brain regions that regulate affective states. One of these regions is the mid brain ventral tegmental area (VTA), a region that contains dopamine neurons critical for the regulation of motivated states.^83^, ^84^, ^85^ These neurons are activated by reinforcers that include palatable foods, as well as sex, social interactions and the intake of drugs of abuse as well as cues that predict the presence of these reinforcers.^83^, ^84^, ^85^

The VTA has also emerged as a region capable of monitoring changes in nutrients and metabolic hormones that include leptin, fatty acids, glucose, insulin, GLP‐1, and ghrelin.^86^, ^87^, ^88^, ^89^, ^90^, ^91^ The release of dopamine in response to these signals has been associated with increased or decreased food seeking and with changes in emotional state.^88^, ^92^, ^93^, ^94^ Much attention has been placed on ghrelin given that ghrelin is the only gastrointestinal hormone that increases appetite.^92^ In the VTA, dopamine cells express the growth hormone secretagogue receptor (GHSR) and respond to ghrelin by increasing action potential frequency and dopamine release.^95^ When ghrelin is delivered into the VTA, mice and rats consume more food and show increased motivation as reflected in increased performance of operant tasks associated with an increased motivated state.^95^, ^96^, ^97^, ^98^, ^99^, ^100^ These responses are attenuated by pretreatment with ghrelin receptor antagonists.^98^, ^99^, ^100^ Ultimately, these data are supportive of ghrelin directly impacting the activity of dopamine neurons to modulate appetite.

There are, however, questions on how ghrelin gets to the VTA to modulate dopamine release, and the mechanistic nature through which ghrelin changes the activity of these cells. Exogenous treatment with ghrelin seems to only influence cells in the AP, NTS, and ARC, and this is potentially because ghrelin access to the brain is restricted to circumventricular regions.^101^, ^102^ Nevertheless, recent work shows that the ability of ghrelin to enter the brain may be related to the metabolic state of the animal. Indeed, there is increased brain permeability to ghrelin and potentially other hormones that can activate regions like the VTA and its target the nucleus accumbens in animals exposed to chronic social defeat stress.^103^ Given that ghrelin has neuroprotective effects, these data would support the hypothesis that under stressful conditions, the blood–brain barrier becomes more permeable to ghrelin and other metabolic hormones, to act on the VTA as a mechanism that sustains motivation for foods that generate positive affect and detection of cues that improve survival.^104^, ^105^

**FIGURE 2:**
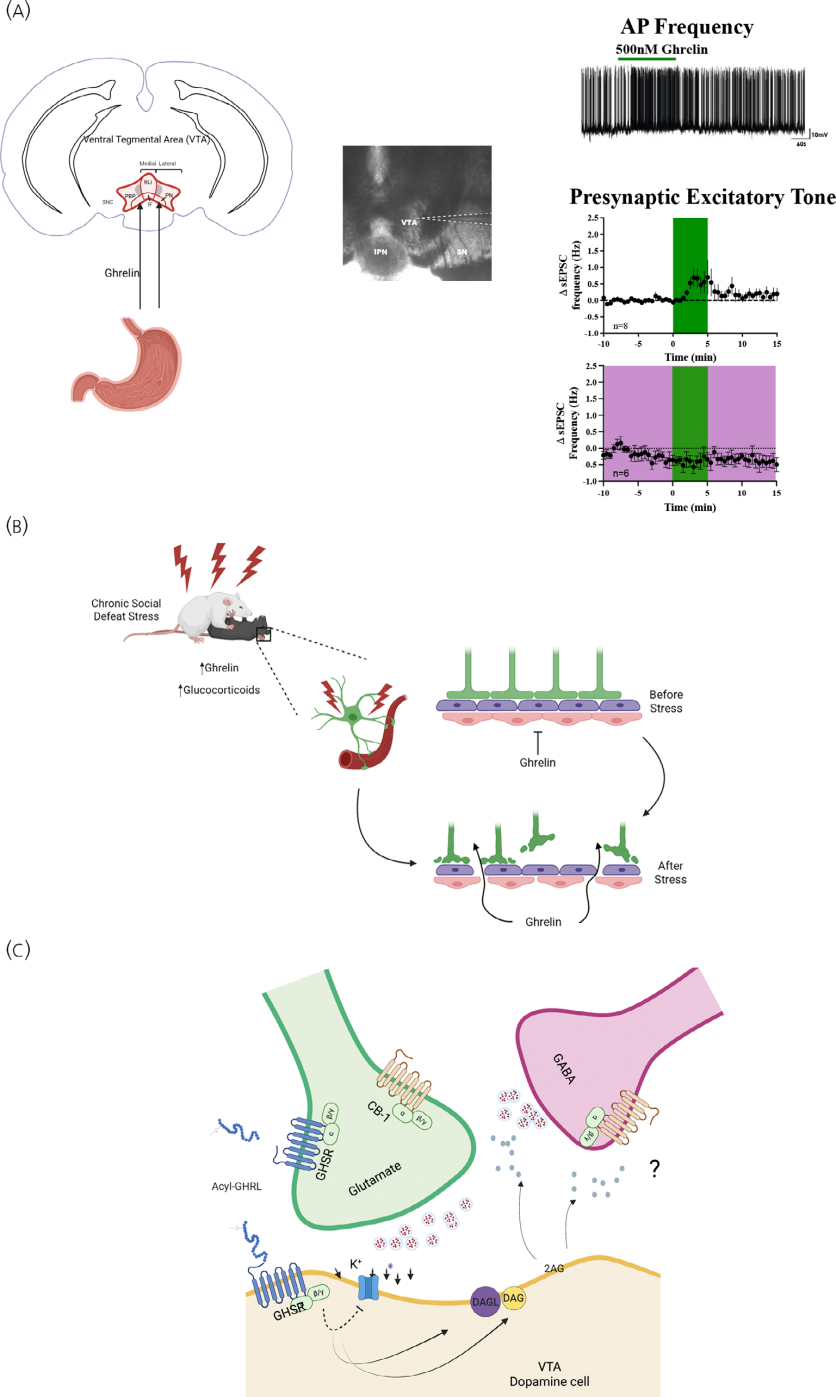
Ghrelin regulation of ventral tegmental area (VTA) dopamine neurons. (A) Ghrelin targets VTA dopamine neurons to stimulate action potential frequency and presynaptic excitatory tone, the latter effect being blocked by pre‐exposure to a cannabinoid receptor (CB‐1r) antagonist (as depicted in the panel filled in pink). (B) Changes in metabolic state produced by chronic stress modify blood–brain barrier permeability to allow more ghrelin to enter the brain and reach the VTA. (C) Once in the VTA, ghrelin may bind to growth hormone secretagogue receptor [GHSR] located postsynaptically to depolarize the membrane and increase action potential frequency. Ghrelin may also directly stimulate glutamate cells, while inhibiting GABAergic tone through the release of 2‐Arachidonylglycerol (2‐AG), although these mechanisms remain to be fully unveiled.

At the level of the cell, the impact of ghrelin on dopamine neurons could involve direct and indirect mechanisms (see Figure 2). This is because the expression of GHSR in the brain occurs in dopamine and non‐dopamine cells.^95^, ^106^ Recent evidence also supports the idea that ghrelin activates dopamine cells directly and non‐dopamine cells indirectly to enhance overall dopamine cell activity. For example, dopamine cells release nitric oxide and endocannabinoids in response to ghrelin, and these can act at the presynaptic level to increase the excitatory tone onto dopaminergic neurons.^107^, ^108^, ^109^ Ultimately, targeting the GHSR in VTA may represent an important target to curb appetite especially in situations where cues that increase food craving are present. In fact, a similar approach has been proposed to treat alcohol abuse with ghrelin receptor antagonists.^110^

The effect of ghrelin on the mesolimbic dopaminergic system may not selectively impact food motivation. There is evidence that ghrelin can promote sex motivation in male mice and rats and decreased GHSR signaling in the VTA reduces sex anticipation in rats.^111^, ^112^ In contrast, infusions of ghrelin into the preoptic area reduced sex anticipation.^111^ Thus, while the actions of ghrelin on these two sites are paradoxical, it is possible that ghrelin acts in the VTA to produce a more generalized state of motivation, while reducing sexual behavior at the level of the hypothalamus to bias behavior towards food and not sex seeking.^111^ There are fewer papers looking at these variables in females, but recent work shows that females may have increased GHSR expression in both the hypothalamus and VTA during the lactation period, and this could reflect an overall increase in drive coming from the VTA, that is biased to increased food intake and maternal care via activation of GHSR in the preoptic area and ARC.^113^

Finally, ghrelin is not the only hormone that can impact the activity of dopamine cells in the VTA. There is evidence that leptin, insulin and GLP‐1 also directly target dopamine cells in the VTA and regulate their activity.^94^, ^114^, ^115^, ^116^ This indicates that homeostatic hormones interact at the level of the VTA to modulate complex processes associated with motivated states including sex motivation. Moreover, the actions of these hormones within the VTA could also be considered targets for the treatment of conditions associated with disruptions in motivated and affective states like addiction and mood disorders.

## 6 SUMMARY

Energy homeostasis is a critical factor that determines investment in costly processes that include reproduction. Recent advances in the field of molecular genetics have allowed for elegant dissections of the systems associated with feeding and reproduction to better understand how these interact to enhance survival of the species. Furthermore, these advances have allowed for a better understanding of how homeostatic systems and systems that regulate cognitive and affective states synergize to produce behaviors like feeding, copulation and parental care. Importantly, such progress has led to the discovery of effective treatments for obesity and infertility, therapies that were only dreamed of a decade ago, but these are not devoid of further challenges. Considering that novel obesity treatments could inadvertently impact reproduction, especially in females, further research is critical. Ultimately, understanding these mechanisms will provide for drug targets that improve human health, especially women's health across different phases of life that include puberty, pregnancy, lactation and reproductive senescence. Furthermore, research on these domains will allow for better prediction of potential side effects associated with obesity treatments and for the design of drugs that avoid these side effects.

## AUTHOR CONTRIBUTIONS

**Naira da Silva Mansano:** Conceptualization; writing – original draft; writing – review and editing. **Calvin Vinh Lieu:** Conceptualization; writing – original draft; writing – review and editing. **Alfonso Abizaid:** Writing – original draft; writing – review and editing; funding acquisition; conceptualization.

## CONFLICT OF INTEREST STATEMENT

The authors declare no conflicts of interest.

## Data Availability

Data sharing not applicable to this article as no datasets were generated or analyzed during the current study.
